# Impact of an AI app‐based exercise program for people with low back pain compared to standard care: A longitudinal cohort‐study

**DOI:** 10.1002/hsr2.1060

**Published:** 2023-01-12

**Authors:** Rica Hartmann, Florian Avermann, Christoff Zalpour, Annika Griefahn

**Affiliations:** ^1^ Faculty Business Management and Social Sciences University of Applied Science Osnabrück Germany; ^2^ medicalmotion GmbH München Germany; ^3^ Institute for Health Sciences Universität zu Lübeck Lübeck Germany

**Keywords:** application, artificial intelligence, exercises, low back pain, mhealth, pain

## Abstract

**Background:**

Low Back Pain (LBP) is a common health problem worldwide. In recent years, the use of mobile applications for the treatment of various diseases has increased, due to the Corona pandemic.

**Objective:**

The aim of this study is to investigate the extent to which artificial intelligence (AI)‐assisted exercise recommendations can reduce pain and pain‐related impairments in daily life for patients with LBP, compared to standard care.

**Methods:**

To answer the research question, an 8‐week app‐based exercise program was conducted in the intervention group. To measure the influence of the exercise program, pain development and pain‐related impairment in daily life have been evaluated. A so‐called rehabilitation sports group served as the control group. The main factors for statistical analysis were factor time and group comparison. For statistical calculations, a mixed analysis of variance for pain development was conducted. A separate check for confounders was made. For pain impairment in daily life nonparametric tests with the mean of change between the time points are conducted.

**Results:**

The intervention group showed a reduction in pain development of 1.4 points compared to an increase of 0.1 points in the control group on the numeric rating scale. There is a significant interaction of time and group for pain development. Regarding pain‐related impairments in daily life, the intervention group has a reduction of the oswestry disability index scores by 3.8 points compared to an increase of 2.3 in the control group. The biggest differences become apparent 8 weeks after the start of treatment. The significant results have a medium to strong effect.

**Conclusion:**

The results shown here suggest that the use of digital AI‐based exercise recommendations in patients with LBP leads to pain reduction and a reduction in pain‐related impairments in daily living compared to traditional group exercise therapy.

## 1 INTRODUCTION

According to the Global Burden of Disease Study 2015, low back pain (LBP) is one of the most prominent and most prevalent diseases worldwide.^1^ LBP covers a spectrum of different pain types, like nociceptive, neuropathic, and/or nociplastic.^2^ Due to that, the clinical presentation is sometimes unclear and may overlap.^2^ Fatoye et al.^3^ summarized that different risk factors promote LBP, for example, depression and psychological distress. Being male or female may also be a factor that requires attention from clinicians and patients themselves. In 2022, spending on LBP in Germany for the ICD diagnosis M54 alone was €3.8 billion, which corresponds to 1% of total healthcare spending.^4^

In various treatment guidelines, nonpharmacological treatments are considered in addition to the implementation of the biopsychosocial model during treatment, and self‐management and active exercise therapy are the focus in addition to education.^5^, ^6^, ^7^ In the presented guideline for managing acute and chronic back pain by George et al.^8^ it is addressed that different clinical representations should also be considered in exercise therapy. That exercise selection must be adjusted accordingly. This is where digitization can come in; the possibility of linking education and exercise therapy with each other makes the use of apps seem sensible.^9^ In a systematic review by Machado et al.^9^ various mobile applications were evaluated, and it became clear that many applications also include parts of the Guideline, but the studies regarding effectiveness are poor. These results are also supported by Du et al.^10^ In their meta‐analysis, the positive aspects of the treatment of LBP could also be presented, yet the clinical evidence is poor.^10^

Nevertheless, positive study results are already available. Through a multidisciplinary back pain app, positive effects on pain reduction numeric rating scale (NRS) as well as on functionality could be detected after 12 weeks, whereby the results were still consistent after 12 months.^11^, ^12^, ^13^, ^14^ Further analyses showed that savings of −80% as well as of 416 Euros per reduced point on the pain index (NRS) were caused by this therapy possibility.^14^

Another digital exercise program was able to achieve an average pain improvement visual analog scale (VAS) of 68.45% between initial measurement and 12 weeks in a wide‐ranging study of over 10,000 subjects, and 78.60% of program completers even achieved a (minimally) important change in pain.^15^ Researchers led by Shebib et al.^16^ report comparable results regarding pain with an average pain reduction of 52%−64% and an average improvement for functional outcomes of 31%−55%. Another digital decision support system showed positive effects after 6 weeks.^17^

Despite these results, the studies do not represent a sufficiently concrete comparison to standard care with conventional therapy methods. Due to this, the following research question was defined “What is the impact of an AI‐ and app‐based exercise program in patients with LBP on pain and pain‐related impairments in daily life compared to standard care?”

## 2 METHOD

The study was conducted in accordance with the ethical principles of the Declaration of Helsinki and in consideration of the STROBE guideline.^18^ The complete STROBE checklist can be found in the appendix. A positive ethical vote was given by the ethics committee of the Osnabrück University of Applied Sciences.

### 2.1 Trial design

A longitudinal cohort study was conducted to answer the previously stated research question. For this purpose, two groups were formed—the intervention group (AI recommended app exercise program) and the control group (usual care as group sport—so called rehabilitation sport in Germany). Randomization, as well as blinding of the subjects, was not possible, since the control group was permanently enrolled in the rehabilitation sports group. The typical biases of a cohort study (selection bias and information bias) were attempted to be addressed at an early stage.^19^

### 2.2 Participants'

The following inclusion criteria were applied: (1) Men and women with back pain, and (2) age between 18 and 65 years with access to the internet and a digital device (smartphone/tablet). Exclusion criteria were: (1) diagnosed neurological or psychological condition, (2) infection or systemic disease, (3) regular medication for mental illness. The sample of this study consisted of all registered participants who fulfilled the inclusion criteria and completed at least the entry questionnaire.

In a first step prospective participants were informed via flyers, posters, social media, and direct approaches in physical therapy practices. In this way, interested study participants received the first information about the planned study. If interested, prospective participants were able to contact the research group via phone or mail. When contacting the research group, the prospective participants were informed about the inclusion and exclusion criteria and open questions could be clarified. Only by contacting the research group, interested participants were able to participate in the study. At the beginning of the study, the participants signed a consent form, which could be revoked at any time without giving reasons.

### 2.3 Interventions

The control group—usual care—received group sport over 12 weeks as part of the so‐called rehabilitation sport. This involved a group session one to two times a week for about 45 min. The exercises included strengthening, stretching, and/or mobilization exercises and are designed to treat and train the complaints in the neck and back areas. The intervention group received recommended exercises via an app with the help of an artificial intelligence (AI), which adapts the exercise recommendations to the input of the user. The intervention group was able to perform a maximum of five exercises daily.

### 2.4 Outcomes

The primary outcome was a pain score using the NRS. The scale describes the pain rating on a scale of 0−10. A change of 2 points is classified as clinically relevant.^20^

Secondary outcomes were pain‐related impairment in daily living, measured with the oswestry disability index (ODI).^21^ The ODI describes the impairment in daily life due to the pain. In the questionnaires, the domains Pain Intensity, Personal Care, Lifting, Reading, Concentration, Work, Driving, Sleeping, and Recreation, among others, are rated on a scale from 0 to 10. A change of 4 points on the ODI is classified as clinically relevant.^22^

Outcomes were reported using an online questionnaire at t0 (baseline), t1 (after 4 weeks), and t2 (after 8 weeks). The questionnaire was preceded by a written declaration of consent regarding the processing of personal data. The initial questionnaire (t0—Baseline measuring) included a query of demographic and general data on age, gender, occupation, daily work routine, and frequency of sporting activities. Red flags and exclusion criteria were determined by means of specific questions and, if necessary, excluded from the study.

### 2.5 Statistical analysis

The aim of the statistical analysis was to investigate effects of an app‐based exercise program compared to usual care in people with back pain over several measurement time points.

For the statistical evaluation, all personal data was anonymized so that no inference could be drawn about the person. Due to fixed conditions (fixed sample size according to rehabilitation sport groups), no sample size calculation was performed previously. Only participants with scores at every time point will be included.

For baseline analysis group comparison will be conducted. For categorical variables absolute numbers, percentages, odds ratios (OR) and lower (l) and upper (u) confidence interval (CI) and for continuous variables mean, standard deviation (SD), standardized mean difference and CI were used. For comparison *χ* ^2^‐test (categorical variables) and independent *t*‐test (continuous variables) or Mann−Whitney *U* Test is conducted.

For longitudinal analysis of the factor pain development a mixed analysis of variance (ANOVA) model is used. Furthermore, an ANOVA with repeated measurements is used for the influence of time. Possible confounder will be separately included in the model and if significant they stay in the model. Mann−Whitney *U* Test for group comparison with the values of change between the time points for the factor pain impairment in daily life are conducted. To analyze the factor time in each group a Wilcoxon Test is conducted.

The evaluation of significance (*p*) was set at *p* = 0.05 or *p* = 0.017 (Bonferroni‐Correction). The effect size will be evaluated with Cohens *d*, based on *z*‐statistics as *r*, or based on the *η*² as f. The interpretation of the effect size is based on Cohen.^23^ All analyses were two‐sided and conducted with the statistical software package of SPSS (version 27) by IBM.

To answer this research question, the following hypotheses were generated and statistically tested as we proceeded.

H1: *There is a significant difference between the intervention group and the control group in terms of pain change measured by the NRS*.
H2: *There is a significant difference between the intervention group and the control group in terms of pain‐related impairment in daily living measured by the ODI*.

### 2.6 Content and description of the medicalmotion app

Medicalmotion GmbH offers a mobile and a web app for users with various kinds of pain and complaints as well as for prevention. In addition to AI‐based exercise recommendations, the mobile app also includes relaxation exercises, podcasts, a chat function, and a health cockpit, where the users can update and track their condition. The AI‐based exercise recommendations consider the data from an anamnestic questioning including for example, several pain attributes (like localization, type, and quality) and lifestyle‐related information, as well as the daily wellbeing and pain sensation. Furthermore, the user's feedback from previously performed exercises is considered in the recommendation process.

Each day, the user can choose the number of exercises to perform (3−5 exercises), which are provided as audio‐guided exercise videos in real‐time. The AI‐based exercise recommendation system chooses the most beneficial exercises out of a pool of 350 exercises by considering the data from an anamnestic questionnaire.

## 3 RESULTS

**Figure 1:**
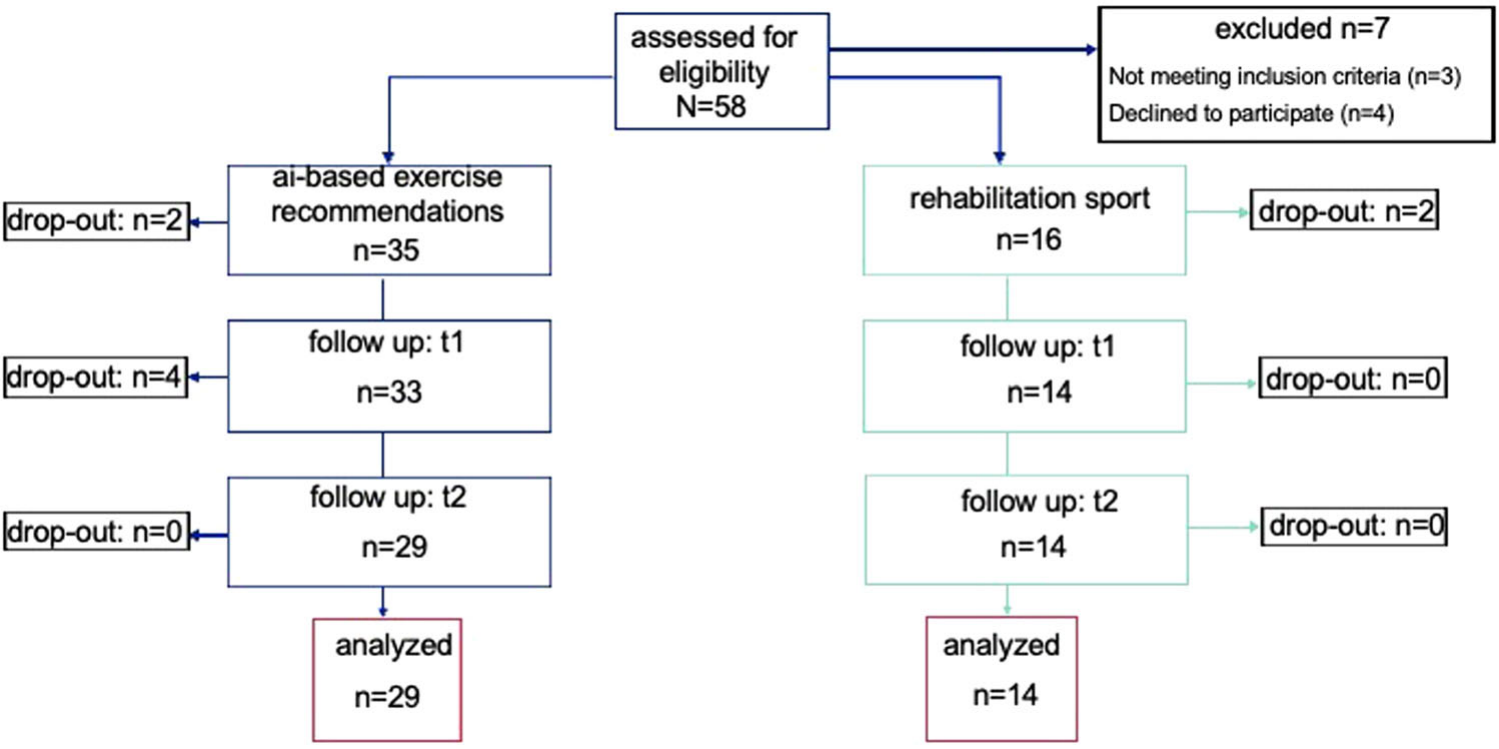
Flow chart

Figure one shows the dropouts at each time point. In total, 58 possible participants were recruited but three did not meet the inclusion criteria and four declined to participate. In the intervention group for back pain, 35 participants met the inclusion criteria. After the three time points, 29 participants were eligible to analyze. After recruitment, 16 participants met the inclusion criteria in the control groups. In total, two participants dropped out, so 14 participants were eligible to analyze (Figure 1).

### 3.1 Sample size

The 58 participants were on average 44.5 (SD 12.3) years old. Most of them had a sitting (44%) or a sitting and standing (44%) working position. Furthermore, 60% do sports one to three times a week.

On average, the members of the intervention group were 41.4 (SD 12.5) years old and most of them do sports one to three times a week (48%). The most frequent working position is sitting (55%). In the intervention group are more female participants (59%). At baseline the pain development score is 5.9 (SD 2) and the pain impairment in daily life score is 15.2 (SD 8.9).

On average, the participants in the control group were 50.9 (SD 9.3) years old and most of them do sports one to three times a week (86%). The most frequent working position is sitting and standing (64%). Male and female gender are equally represented in the control group. At baseline the pain development score in the control group is 6.3 (SD 1.6) and the pain impairment in daily life score is 22.7 (SD 5).

At baseline sports duration differs between control group and intervention group significantly in following durations: never (*p* = 0.008) and one to three times (*p* = 0.02). The biggest difference is that 11 people in the intervention group reported not doing any sports, to no one in the control group. The working position “sitting” (*p* = 0.04) is also significantly different. The mean age of the control group is 50.9 years and for the intervention group 41.4 years (*p* = 0.02).

At every time point, the control group has significantly higher scores in the ODI questionnaire compared to the intervention group. Despite at baseline similar result is shown for pain scores at 4 weeks and 8 weeks. The control group reported higher scores at those time points. Table 1 shows the descriptive statistics at baseline and the value for pain and pain‐related impairment in daily living over time divided by groups.

**Table 1:** Descriptive statistics for intervention and control group

	Intervention group (App usage) *n* = 29	Control group (Reha sport) *n* = 14		
Categorical variables	Absolute numbers (%)	Absolute numbers (%)	OR (lCI; uCI)	*p*
Sex (male/female)	12/17 (41)	7/7 (50)	0.7 (0.2−2.5)	0.59
Typical working position				
Sitting	16 (55)	3 (21)	0.2 (0.1−1)	0.04
Sitting + standing	10 (35)	9 (64)	3.4 (0.9−13)	0.07
Standing	2 (7)	1 (7)	1 (0.1−12.5)	0.98
Physically hard work	1 (3)	1 (7)	2.2 (0.1−37.2)	0.59
Sport duration (frequency per week)				
Never	11 (38)	0	0.6 (0.4−0.8)	0.008
1 to 3 times	14 (48)	12 (86)	6.4 (1.2−34)	0.02
More than 3 times	4 (14)	2 (14)	1 (0.2−6.5)	0.97

Continuous variables	Mean (SD)	Mean (SD)	SMD (lCI; uCI)	*P*
Age	41.4 (12.5)	50.9 (9.3)	−0.8 (−1.5 to –0.2)	0.02
Pain development				
Baseline	5.9 (2)	6.3 (1.6)	−1 (−0.9 to 0.4)	0.45
After 4 weeks	4.8 (2.3)	6.4 (1.6)	−0.8 (−1.5 to –0.1)	**0.02**
After 8 weeks	4.5 (2)	6.4 (1.6)	−1 (−1.7 to –0.3)	0.003
Pain impairment in daily life				
Baseline	15.2 (8.9)	22.7 (5)	−1 (−1.6 to –0.3)	0.005
After 4 weeks	13 (6.7)	22.1 (4.7)	−1.5 (−2.2 to –0.8)	**<0.001**
After 8 weeks	11.4 (7.7)	25 (12.3)	−1.4 (−2.2 to –0.7)	**<0.001**

*Note*: Bold values are statistically significant.

### 3.2 Pain development

The intervention group showed a change on the NRS from 5.9 (t0) to 4.79 (t1) and 4.5 (t2). This represents a reduction of 1.4 points. The control group showed a change on the NRS from 6.3 (t0), 6.4 (t1) to 6.4 (t2). This represents an increase of 0.1 points. Statistical analysis showed no significant effect at baseline (*p* = 0.45), after 4 weeks (*p* = 0.02) and significant differences after 8 weeks (*p* = 0.003) when comparing between groups.

There was a statistically significant interaction between time and group, *F* (2, 82) = 3.791, *p* = 0.03, partial *η*² = 0.085. The significant difference after eight weeks has a strong effect size of 1 (Cohens). The possible confounders age (*F* (1, 39) = 1.51, *p* = 0.23, partial *η*² = 0.037), sex (*F* (1, 39) = 0.21, *p* = 0.65, partial *η*² = 0.005), sport duration (*F* (2, 38) = 1.17, *p* = 0.32, partial *η*² = 0.058) and work situation (*F* (3, 35) = 1.53, *p* = 0.22, partial *η*² = 0.116) will not be included in the model. In Table 2a the statistical analysis for the independent *t*‐test is summarized.

**Table 2a:** Independent *t*‐test for pain development

Time points	Mean difference	Standard error difference	lCI; uCI	*p*	Cohens *d*
Baseline	−0.5	0.6	−1.7 to 0.8	0.45	0.3
After 4 weeks	−1.6	0.7	−3 to 0.3	0.02	0.8
After 8 weeks	−1.9	0.6	−3.1 to 0.7	0.003	1

The factor of time is highly significant in the intervention group, *F* (2, 56) = 7.47, *p* = 0.001, partial *η*² = 0.211. In comparison the control group has no significant result, *F* (2, 26) = 0.14, *p* = 0.87, partial *η*² = 0.011. Based on the *η*² the effect size *f* is 0.89 which indicates a strong effect.^23^ In Table 2b the statistical data for the ANOVA with repeated measurements is presented.

**Table 2b:** ANOVA with repeated measurements (intervention group)

Time points	Mean difference	lCI; uCI	*p*
Baseline to 4 weeks	1.1	0.3−1.9	0.007
Baseline to 8 weeks	1.4	0.4−2.3	0.004
4 weeks to 8 weeks	0.3	−0.7 to 1.3	>0.99

Abbreviation: ANOVA, analysis of variance.

**Figure 2:**
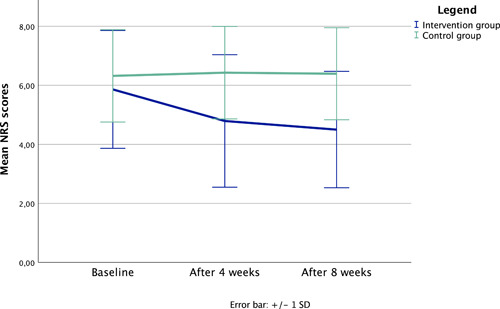
Pain development over time—line chart

As seen in Figure 2 the intervention group has a reduction of 1.4 points on the NRS scale over 8 weeks. The control group has an increase on the NRS of 0.1 points in the same time frame.

### 3.3 Pain impairment in daily life

The intervention group showed a change on the ODI from 15.2 (t0), 13 (t1) to 11.4 (t2). This represents a reduction of 3.8 points. Whereas the control group showed a change from 22.7 (t0), 22.1 (t1) to 25 (t2). This represents an increase of 2.3 points. Statistical analysis showed a significant effect at t0 (*p* = 0.005), t1 (*p* < 0.001) and significant difference at t2 (*p* < 0.001) when comparing between groups.

There is a statistically relevant difference between groups, if we look at the scores at each time point. If you look at the score of change, the group comparison showed no significant differences. Within the intervention group, the factor time seems to have an influence. The difference from baseline to 8 weeks shows a significant result with *p* = 0.008. The effect size *r* ranges between small to medium.^23^ In Table 3 the statistical analysis (Wilcoxon and Mann−Whitney *U* test) for pain impairment in daily life is presented.

**Table 3:** Statistical Analysis of the ODI

Time points	Wilcoxon‐test				Mann−Whitney *U* test	
	Intervention group		Control group			
	*p*	Effect size *r*	*p*	Effect size *r*	*p*	Effect size *r*
Baseline to 4 weeks	0.1	0.3	1	‐	0.27	0.2
Baseline to 8 weeks	0.008	0.5	0.86	0.1	0.08	0.3
4 weeks to 8 weeks	0.13	0.3	0.67	0.1	0.28	0.2

**Figure 3:**
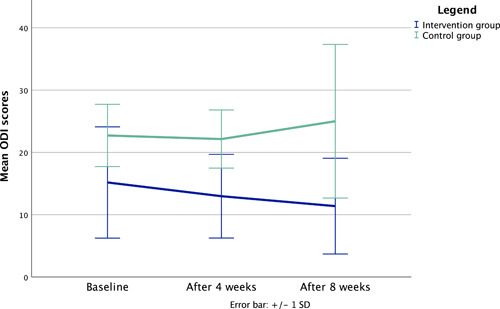
Pain impairment in daily life—line chart

As seen in Figure 3, the pain impairment in daily life described by the mean of the ODI scores reduces by 3.8 in the intervention group compared to an increase by 2.3 in the control group after 8 weeks. The significant reduction after 8 weeks in the intervention group has a medium effect size.

## 4 DISCUSSION

The purpose of this longitudinal study was to investigate the extent to which an app‐based exercise program can change pain intensity as well as pain‐related impairment in daily living in patients with back pain compared to standard care. To answer the research questions, hypotheses were first defined. The following hypothesis was defined to evaluate pain development: *There is a significant difference between the intervention group and the control group in terms of pain change measured by the NRS*. The defined hypothesis could be accepted because a significant difference between the intervention and control groups could be statistically demonstrated (*p* = 0.003 at t2). Nevertheless, the reduction of 1.4 points in the intervention group and the increase of 0.1 points in the control group showed no clinical effect. The clinical effect defined by studies is defined to a change of 2 points.^20^ Similarly, minimal detectable change (MDC) is 1.33 points on the NRS.^20^ The calculation of the effect size (*f*) nevertheless showed a strong effect with *f* = 0.44.^23^ So, the included factors time and group seems to be important for the change in pain development. Nevertheless, further studies with those two factors should be conducted to reduce the risk for possible change by odds and to find not only clinical important factors for pain development but also clinical important changes in the NRS.

To be able to further analyze the difference in pain impairment in everyday life, a hypothesis was also defined for this: *There is a significant difference between the intervention group and the control group in terms of pain‐related impairment in daily living measured by the ODI*. The defined hypothesis could be accepted because a significant difference between the intervention and control groups could be statistically demonstrated (*p* < 0.001 at t2). The intervention group showed a reduction of 3.8 points, whereas the control group showed an increase of 2.3 points. In contrast to this the literature define a clinically relevant result with a change of 4 points on the ODI.^22^ Accordingly, the change within the intervention group bordered on a clinically relevant outcome. In contrast, the control group showed an increase of 2.3 points. The calculation of the effect size (*f*) showed a medium effect with *f* = 0.25.^23^ The usage time seems to be an important part for the changes of pain impairment in daily life only for the intervention group and is not merely statistical relevant. In addition, further research is important to reduce the risk for confounding because the data only allowed weaker statistically calculation. From the previously shown results, the research question can be positively applied.

The studies conducted to date currently vary considerably in a wide range of parameters. For example, the number of subjects varies from 27^24^ to 10,000,^15^ and the outcomes vary from the pure measurement of pain^12^ to the measurement of mental health (e.g., PHQ‐8).^16^ Nevertheless, positive effects are attributed to digital exercise treatments, either with educational component^15^, ^17^ or without.^25^

These positive effects were demonstrated by Zheng et al.^26^ once on pain (NRS) and function (Roland and Morris Disability Questionnaire; RMDQ) using m‐Health‐based core stability exercises after 4 and 16 weeks, with patients who additionally performed self‐compassion training achieving these effects earlier. Two other studies of a mobile app delivering multidisciplinary treatment for pain‐related LBP found the app had a positive effect and a stronger pain reduction on VAS pain compared to the control group after 12 weeks.^12^, ^13^ In contrast to this study, in which the app group differed significantly from the control group in terms of ODI at 4 and 8 weeks, subjects in the app group were only superior to those in the control group in the study by Priebe et al.^13^ in functional outcomes. Besides the positive results in pain development in a study by Priebe et al.^14^ a cost‐effectiveness analysis demonstrated a reduction in health care costs of 416,‐ € per point reduced on the NRS. Given these results, it could be hypothesized that mobile applications also have a cost advantage over conventional rehabilitation exercises. This could be analyzed in future research.

Other authors around Chhabra et al.^27^ and Shebib et al.^16^ were able to demonstrate positive effects on pain and functional abilities using m‐Health programs, as in this study, although in the study by Chhabra et al.^27^ both groups improved significantly, but the app group showed a stronger decline in terms of functional ability. Strikingly, the researchers were only able to demonstrate these results about a group with written prescription and a normal physical activity.^16^, ^27^ Significant improvements in ODI using app‐based exercise were also reported in the study by Hasenöhrl et al. (2020), with baseline ODI decreasing from 17.11% to 14.44% after 4 weeks.^24^ In contrast to the present study, these results were achieved only asides from a control intervention, underscoring the value of an app‐based approach.^24^

It is notable that multiple research projects have already been conducted on the given topic around digital exercise programs in relation to LBP. Nevertheless, the different app concepts also in terms of, for example, exercise type, frequency, and design, as well as the different measurement tools, make it difficult to compare results with those of other studies. They can also differ from each other, for instance, to such an extreme that different results emerge already due to the type of app. The app presented here is based on artificial intelligence, so that patients are offered individual exercises based on their data, which is a considerable advantage over other app concepts without an AI‐based exercise program.

Furthermore, it is obvious that only a few studies are available that draw a comparison to standard care. Often, rather passive measures such as reading of educational material are considered, which also means that in this case no sufficient comparison can be drawn to results of this study. The disadvantage, however, is the lack of a long‐term view, which is offered, for example, by the study of Zheng et al.^26^ Thus, it would be exciting to investigate whether the obtained results of both groups continue to be consistent or indicate a change after a period of 8 weeks. Due to this, further studies are necessary in the future to verify and, if necessary, confirm the stated assumptions by employing similar conditions.

### 4.1 Limitations

In addition to the increasing number of clinical studies regarding effectiveness (pain reduction, reduction of impairments in daily life, etc.), studies regarding risk assessments should not be ignored. For example, Jain et al.^28^ conducted a study on this topic. They found that only 0.00014 adverse events (e.g., increase in pain) were recorded per active day within the app.^28^ However, this was not considered negative because the adverse events were associated with the normal changes of LBP patients.^28^

The results obtained in the longitudinal cohort study conducted here show a positive trend, especially when compared to regular care for back pain. In addition to the lack of randomization as well as the not reported number of exercises may also affect the results. Similarly, the difference in the number of respective subjects in the groups could significantly influence the results. For further studies, randomization and/or case numbers should be increased or adjusted. Another limitation of the study carried out here was that the control group was not asked about the average participation in rehabilitation sports. As a result, no comparison between app use and participation in rehabilitation could be analyzed. Since not only active exercises can have a positive influence on the treatment of LBP, but it would also be interesting for future studies to investigate other functions within the mobile application, such as whether the number of performed relaxation exercises to leads to a higher pain reduction. Through the components of mental health as well as exercise anxiety should also be included in future studies in outcomes in this direction, as these two components may also influence the clinical picture LBP.

## 5 CONCLUSION

This study investigated the impact an AI app‐based exercise program may have on pain and pain‐related impairments compared to so‐called rehabilitation exercise in patients with LBP. Significant results were shown for the group comparison after eight weeks as well as the comparison in the intervention group from t0 to t2 in terms of pain reduction and reduction of pain‐related impairments in daily living.

With the help of these results, it has been shown that a digital health application could be a very promising alternative to normal rehabilitation sport. We therefore recommend that serious consideration be given to the expansion of digital care services in the existing health system, such as the integration of digital solutions in physiotherapeutic care or in the rehabilitation process. In summary, it can be concluded that the use of digital solutions can improve patient reported outcomes and can be an alternative for existing and established solutions in the health care system.

## AUTHOR CONTRIBUTIONS

All authors have read and approved the final version of the manuscript. Annika Griefahn had full access to all of the data in this study and takes complete responsibility for the integrity of the data and the accuracy of the data analysis.

## CONFLICTS OF INTEREST

A. G. and F. A. are employees of medicalmotion GmbH. The remaining authors declare no conflict of interest. The access codes were provided by medicalmotion GmbH to the University of Applied Sciences Osnabrück for the purpose of conducting the study.

## TRANSPARENCY STATEMENT

The lead author Annika Griefahn affirms that this manuscript is an honest, accurate, and transparent account of the study being reported; that no important aspects of the study have been omitted; and that any discrepancies from the study as planned (and, if relevant, registered) have been explained.

## Supporting information

Supporting information.

## Data Availability

The datasets used and/or analyzed during the current study are available from the corresponding author on reasonable request.
